# RABL6A Regulates Schwann Cell Senescence in an RB1-Dependent Manner

**DOI:** 10.3390/ijms22105367

**Published:** 2021-05-20

**Authors:** Jordan L. Kohlmeyer, Courtney A. Kaemmer, Shaikamjad Umesalma, Francoise A. Gourronc, Aloysius J. Klingelhutz, Dawn E. Quelle

**Affiliations:** 1 The Department of Neuroscience and Pharmacology, Carver College of Medicine, University of Iowa, Iowa City, IA 52242, USA; jordan-kohlmeyer@uiowa.edu (J.L.K.); courtney-waite@uiowa.edu (C.A.K.); umesalma-shaikamjad@uiowa.edu (S.U.); 2 The Department of Microbiology and Immunology, Carver College of Medicine, University of Iowa, Iowa City, IA 52242, USA; francoise-gourronc@uiowa.edu (F.A.G.); al-klingelhutz@uiowa.edu (A.J.K.); 3 The Department of Pathology, Carver College of Medicine, University of Iowa, Iowa City, IA 52242, USA

**Keywords:** RABL6A, retinoblastoma protein (RB1), Schwann cell, senescence, MPNST

## Abstract

Schwann cells are normally quiescent, myelinating glia cells of the peripheral nervous system. Their aberrant proliferation and transformation underlie the development of benign tumors (neurofibromas) as well as deadly malignant peripheral nerve sheath tumors (MPNSTs). We discovered a new driver of MPNSTs, an oncogenic GTPase named RABL6A, that functions in part by inhibiting the RB1 tumor suppressor. RB1 is a key mediator of cellular senescence, a permanent withdrawal from the cell cycle that protects against cell immortalization and transformation. Based on the RABL6A-RB1 link in MPNSTs, we explored the hypothesis that RABL6A promotes Schwann cell proliferation and abrogates their senescence by inhibiting RB1. Using sequentially passaged normal human Schwann cells (NHSCs), we found that the induction of replicative senescence was associated with reduced expression of endogenous RABL6A. Silencing RABL6A in low passage NHSCs caused premature stress-induced senescence, which was largely rescued by co-depletion of RB1. Consistent with those findings, *Rabl6*-deficient MEFs displayed impaired proliferation and accelerated senescence compared to wildtype MEFs. These results demonstrate that RABL6A is required for maintenance of proper Schwann cell proliferation and imply that aberrantly high RABL6A expression may facilitate malignant transformation.

## 1. Introduction

Schwann cells are key components of the peripheral nervous system that surround and insulate nerve axons by producing a myelin sheath [1]. Schwann cells halt proliferation after surrounding axons in a one-to-one ratio; however, nerve injury or oncogenic signals induce dedifferentiation and stimulate cell cycle reentry. The Schwann cell lineage gives rise to multiple neoplastic lesions, including neurofibromas (NFs), schwannomas, and malignant peripheral nerve sheath tumors (MPNSTs) [2]. Both NFs and MPNSTs are linked to the common neurological disease called Neurofibromatosis Type I (NF1) [3]. Patients inherit germline mutations in the *NF1* gene, which encodes the Ras GTPase-activating protein neurofibromin. Loss of *NF1* leads to unrestrained Ras signaling and cell hyperproliferation, ultimately culminating in tumor development. 

Although Ras signaling remains “on” in *NF1* mutant cells, neurofibromas are benign and eventually stop proliferating due to the induction of oncogene-induced senescence (OIS). Cellular senescence was first recognized as a permanent exit from the cell cycle by Hayflick and Moorhead in 1961 [4]. They extensively passaged human cells in culture and found that normal cells have a limited, finite lifespan. Numerous studies have delineated the molecular basis for senescence, which is complex and triggered by multiple mechanisms including telomere shortening (termed replicative senescence), accumulation of DNA damage, and upregulation of the *CDKN2A* (also called *INK4A/ARF*) locus, which encodes two powerful tumor suppressors, p16INK4a and the Alternative Reading Frame (ARF) protein [5]. The *INK4A/ARF* locus is inactivated in the majority of human cancers [6]. Paradoxically, hyper-proliferative signaling, such as that resulting from abnormal Ras expression or activation, induces an indistinguishable arrest called OIS [7]. Another type of telomere-independent senescence, termed stress-induced senescence, occurs in response to sub-cytotoxic stress with responses mimicking those observed in replicative senescence and OIS [8,9]. While each form of senescence may result from different stress stimuli and pathways, they share fundamental biological features including resistance to apoptosis and irreversible growth arrest.

Two essential mediators of senescence are the protein products of the *INK4A/ARF* locus. p16INK4a activates the retinoblastoma (RB1) protein by inhibiting cyclin-dependent kinases 4 and 6 (CDK4/6), resulting in the accumulation of hypo-phosphorylated RB1 that enforces senescence [5,6]. By comparison, ARF (p14 in humans, p19 in mice) induces senescence through its activation of p53 and other antiproliferative factors [5,6]. Engagement of the p16INK4a-RB1 and ARF-p53 tumor suppressive pathways prevents Schwann cell proliferation after nerve injury or oncogene activation [10]. Unfortunately, a fraction of these cells can escape senescence by inactivating *INK4A/ARF*, leading to the formation of benign, incompletely transformed lesions called atypical neurofibromatous neoplasms of uncertain biological potential (ANNUBPs). Over time, additional mutations accumulate in the ANNUBPs to cause full transformation into highly aggressive, deadly sarcomas called malignant peripheral nerve sheath tumors (MPNSTs) [11]. 

RABL6A (also called Parf, RBEL1, and *c9orf86*), is an oncogenic GTPase involved in the pathogenesis of numerous human cancers, including MPNST [12]. RABL6A regulates multiple central pathways in tumorigenesis. Specifically, RABL6A has been found to promote tumor cell proliferation and survival by activating oncogenic factors such as ERK [13,14], AKT [15], and MDM2 [16] while inactivating tumor suppressive PP2A [15], p53 [16,17], and p27-RB1 [17,18] to control tumor cell proliferation and survival. We recently discovered that RABL6A promotes MPNST development in part through its activation of CDKs and inhibition of RB1 [12]. This was consistent with our analyses of human tumors revealing RABL6A expression is low in benign NFs and increases through the stepwise transformation process from NFs to ANNUBPs to MPNSTs. Interestingly, while cultured MPNST cells were found to be addicted to RABL6A for their survival, loss of RABL6A in non-transformed Schwann cells caused cell cycle arrest without death [12]. Those results suggested RABL6A might affect the senescence process. Because RB1 induction is critical to all forms of senescence, and RABL6A antagonizes RB1, this study sought to define the role of RABL6A in controlling Schwann cell senescence. 

Here, we establish an essential role for RABL6A in regulating senescence in primary normal human Schwann cells (NHSCs) and mouse embryonic fibroblasts (MEFs). Endogenous RABL6A expression inversely correlated with established markers of cellular senescence including p16INK4a, RB1 hypo-phosphorylation, and senescence-associated β-galactosidase activity. Importantly, RABL6A silencing in low-passage NHSCs caused premature stress-induced senescence, which was attenuated by co-depletion of RB1. In agreement with those findings, *Rabl6*-deficient MEFs displayed impaired proliferation and accelerated senescence compared to wildtype *Rabl6+/+* MEFs. Our results highlight that normal levels of RABL6A are required for maintenance of proper Schwann cell biology and proliferation.

## 2. Results

### 2.1. RABL6A Expression Inversely Correlates with Markers of Cellular Senescence in Primary NHSCs

**Figure 1:**
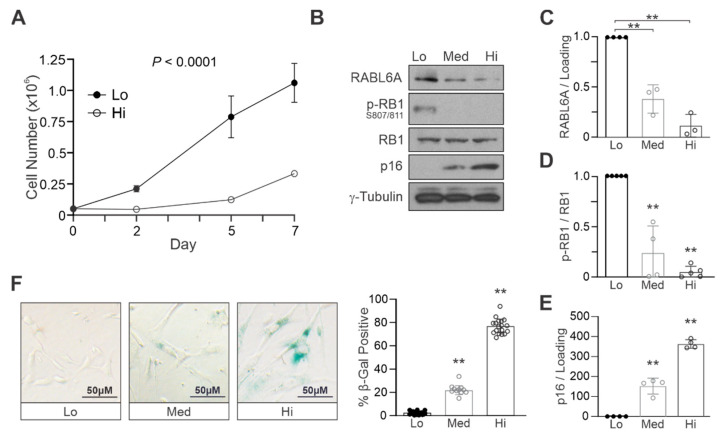
RABL6A expression inversely correlates with markers of cellular senescence in primary NHSCs. (**A**) Low (Lo) versus high (Hi) passage NHSCs were plated at equal density, and cell number was counted on days 2, 5, and 7 after plating. Lo, P2–4; Hi, P8–10. (**B**) Representative Western blot shows RABL6A expression decreases with sequential passaging of NHSCs, which inversely correlates with markers of cellular senescence (reduced phosphorylation of RB1 and induction of p16). Med, medium passage cells (P5–7). ImageJ quantification of western data from three of more experiments showing (**C**) reduced RABL6A expression, (**D**) reduced phopho-RB1 S807/811, and (**E**) increased p16 expression in Lo, medium (Med), and Hi passage NHSCs. (**F**) Left, representative bright field images of β-galactosidase staining, showing an increase in senescent cells upon sequential passaging (Lo, Med and Hi). Right, quantification from three or more experiments, each performed in duplicate. Each dot represents a minimum of 150 cell counts per well. (**A**) *p* value determined by a generalized linear model to assess the difference between the curves. Error bars, SD from mean. (**C**–**F**) *p* value, One-way ANOVA with Dunnett’s correction. (**, *p* < 0.001).

To begin defining RABL6A’s role in Schwann cell senescence, we first examined its expression in primary normal human Schwann cells (NHSCs) at various times during sequential passaging relative to markers of senescence (Figure 1). NHSCs were harvested at low (P2–4), medium (P5–7), and high (P8–10) passages. As passage number increased, the proliferative capacity of the cells was reduced, as shown by the near absence of growth for high passage cells (Figure 1A). Interestingly, endogenous RABL6A expression decreased significantly as NHSCs were passaged and underwent senescence (Figure 1B,C). This correlated with expected changes in molecular and biological markers of senescence. Specifically, p16INK4a (p16) was upregulated with increasing passage, while RB1 phosphorylation at S807/811, sites of CDK4/6 phosphorylation, was reduced (Figure 1B,D,E). A concomitant induction of senescence-associated β-galactosidase (SA-βgal) was also observed (Figure 1F). These data demonstrate that RABL6A levels are normally downregulated during NHSC senescence. 

### 2.2. Loss of RABL6A Causes Premature Stress-Induced Senescence in Primary NHSCs

**Figure 2:**
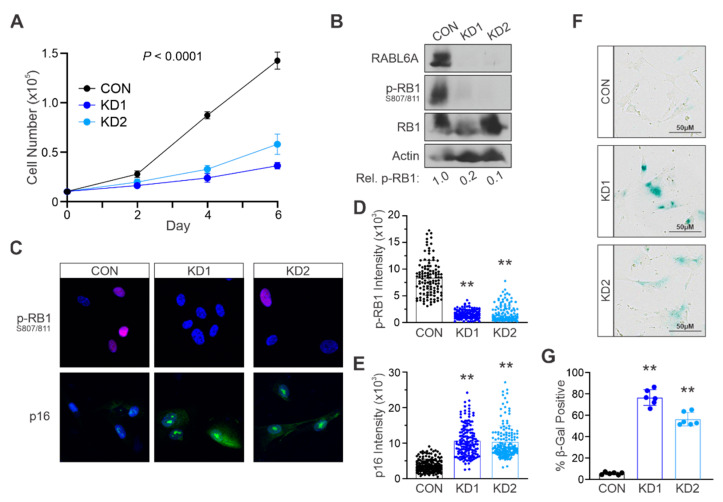
Loss of RABL6A causes premature stress-induced senescence in primary NHSCs. (**A**) Low passage NHSCs with control or *RABL6A* knockdown (KD1 and KD2) were plated at equal density and cell number counted on days 2, 4, and 6 after plating. (**B**) Representative Western blot showing effective RABL6A knockdown in KD1 and KD2 and decreased phospho-RB1 S807/811 compared to CON. Below, quantified relative p-RB1 to total RB1. (**C**) Representative phospho-RB1 S807/811 (top, red) and p16 (bottom, green) immunofluorescence images shows RABL6A depletion causes reduced phospho-RB1 S807/S811 and induction of p16 compared to control cells. Nuclei stained with DAPI. ImageJ quantification of fluorescent intensity from three or more experiments of (**D**) phospho-RB1 S807/811 and (**E**) p16. Each dot in D and E represents an individual cell nuclei. (**F**) Representative bright field images of β-galactosidase staining, showing RABL6A loss induces senescence in NHSCs. (**G**) Quantification from three or more experiments of β-galactosidase positive cells after RABL6A knockdown. (**A**) *p* value determined by a generalized linear model to assess the difference between the KD1 and KD2 curves compared to CON. Error bars, SD from mean. (**D**,**E**,**G**) *p* value, One-way ANOVA with Dunnett’s correction. (**, *p* < 0.001).

To test the importance of RABL6A in NHSC senescence, the endogenous protein was knocked down in low passage cells. Two different short hairpin RNAs (shRNA), named KD1 and KD2 [13,17], were employed, which we recently showed induced a cell cycle arrest in NHSCs without causing cell death [12]. Compared to control (CON) NHSCs expressing empty shRNA vector, cells lacking RABL6A proliferated significantly slower (Figure 2A). Loss of RABL6A led to a dramatic reduction in phosphorylated RB1-S807/811 as well as stark upregulation of p16 expression. This was shown by both Western blotting (Figure 2B) and immunofluorescent (IF) staining of each protein in individual cells (Figure 2C–E). Notably, IF became a valuable approach for assessing molecular changes in these studies since RABL6A loss caused such a significant decrease in cell number. 

Consistent with the above results, low passage NHSCs depleted of RABL6A displayed a significant upregulation of SA-βgal staining (Figure 2F,G). These data show that loss of RABL6A exerts a non-lethal arrest in NHSCs mimicking stress-induced senescence [8]. Thus, RABL6A is required for NHSC proliferation while its absence provokes senescence.

### 2.3. RABL6A Promotes NHSC Proliferation and Evasion of Senescence via RB1 Inactivation

RABL6A promotes the proliferation and survival of several cancers, including MPNSTs, by negatively regulating RB1 [12,17,18]. Since RB1 is a key regulator of cellular senescence that is activated upon RABL6A loss in NHSCs, we silenced RB1 in proliferating NHSCs to test its importance to the RABL6A knockdown phenotype of premature stress-induced senescence. 

**Figure 3:**
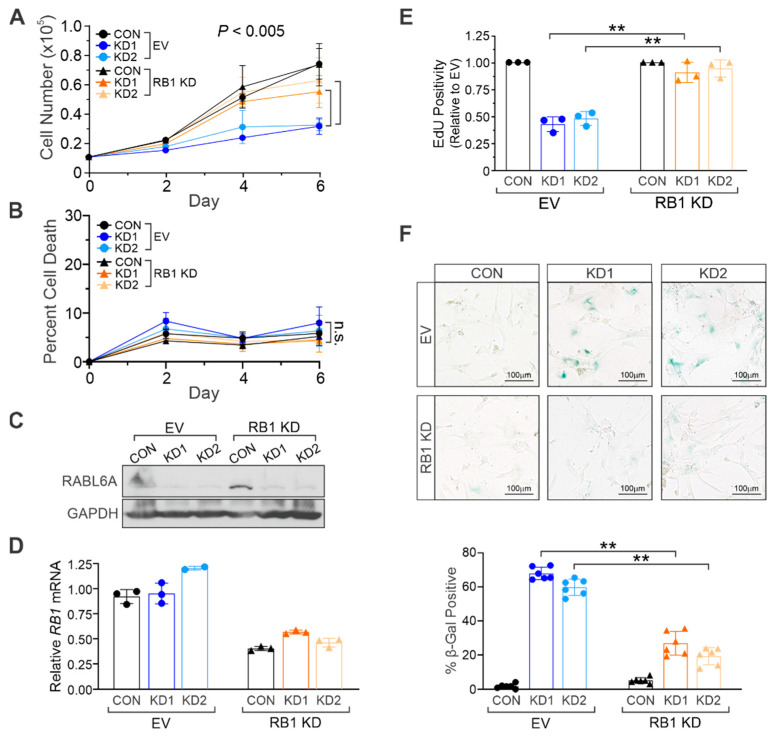
RABL6A promotes NHSC proliferation via RB1 inactivation. Control (CON) and RABL6A knockdown (KD1 and KD2) in low passage empty vector (EV) and RB1 knockdown (RB1 KD) cells were plated at equal density, and (**A**) cell number or (**B**) percent of cell death in the population (as measured by trypan blue exclusion) was counted days 2, 4, and 6 after plating. (**C**) Representative Western blot shows efficient knockdown of RABL6A in KD1 and KD2 samples relative to CON. (**D**) Relative mRNA levels of RB1, demonstrating knockdown of RB1 in RB1 KD samples. (**E**) Quantification of EdU positivity showing RB1 loss restores proliferation in cells with RABL6A knockdown. (**F**) Representative bright field images of β-galactosidase staining, showing the senescence caused by RABL6A loss is attenuated with co-depletion of RB1. Below, quantification of β-galactosidase staining from three experiments performed in duplicate. (**A**,**B**) *p* value determined by a generalized linear model to assess the difference between CON KD1 vs. RB1 KD1 and CON KD2 vs. RB1 KD2 (as indicated by brackets). Error bars, SD from mean. (**E**,**F**) *p* value, Two-way ANOVA with Bonferroni correction. (**, *p* < 0.001).

Low passage NHSCs were sequentially infected with RB1 shRNA (RB1 KD) or empty vector (EV) viruses followed by CON or RABL6A-targeted KD1 and KD2 lentiviruses. As anticipated, loss of RABL6A alone reduced NHSC proliferation compared to CON cells (Figure 3A). RB1 loss alone had no effect on proliferation, whereas it partially rescued the anti-proliferative effects of RABL6A knockdown. Importantly, depletion of RABL6A had no significant effects on NHSC death regardless of RB1 status (Figure 3B). Western blot analyses confirmed effective knockdown of RABL6A, and RT-qPCR confirmed RB1 silencing (Figure 3C,D). Consistent with the growth curve analyses, individual loss of RABL6A caused NHSC cell cycle arrest, as measured by diminished EdU incorporation (Figure 3E), which was coincident with greatly increased SA-βgal activity indicative of senescence (Figure 3F). Remarkably, loss of RB1 in RABL6A depleted cells restored DNA synthesis in NHSCs to control cell levels (Figure 3E) and dramatically reduced the induction of senescence (Figure 3F). These results demonstrate that RABL6A promotes NHSC cell cycle progression and evasion of senescence through RB1 inactivation. 

### 2.4. RABL6A Loss in Mouse Embryonic Fibroblasts Reduces Proliferative Capacity and Causes Premature Senescence

**Figure 4:**
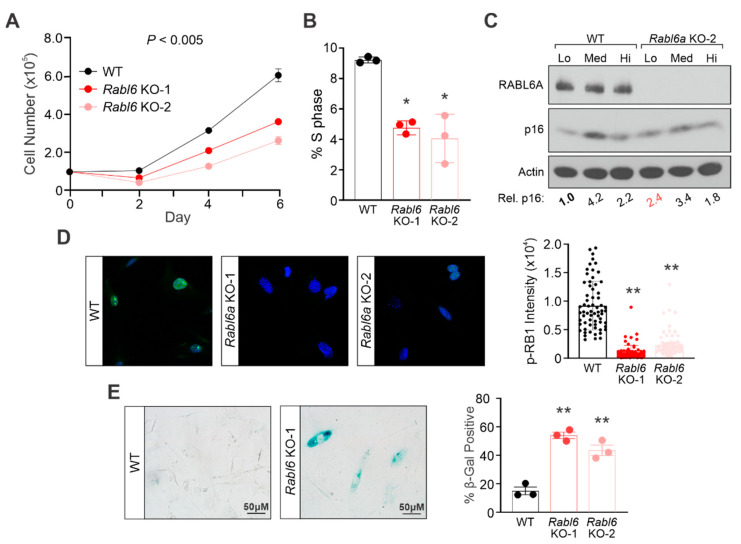
Rabl6a-deficiency leads to reduced proliferative capacity in mouse embryonic fibroblasts. MEFs were harvested from wildtype (WT) or Rabl6 knockout (KO) mice and analyzed. (**A**) Growth curves from MEFs plated at equally low density and counted on days 2, 4, and 6 after plating. (**B**) Flow cytometric analyses of DNA content show a reduction in S phase in asynchronously growing Rabl6a KO MEFs as compared to WT. (**C**) Representative Western blot of sequentially passaged WT versus Rabl6 KO MEFs showing cells lacking RABL6A display elevated p16, a marker of senescence, earlier than WT. Below, quantified p16 levels from ImageJ densitometry analysis. (**D**) Representative immunofluorescence images of phospho-RB1 S807/811 (green) in low passage WT and Rabl6a KO cells, showing cells lacking Rabl6 activate RB1 earlier than WT. Nuclei stained with DAPI. Right, ImageJ quantification of fluorescent intensity of p-RB1. Dots represent individual cell nuclei. (**E**) Representative bright field images of β-galactosidase staining at the same passage number. Right, quantification from three or more experiments of β-galactosidase positive cells, showing an increase in Rabl6a knockout MEFs versus WT. (**A**) *p* value determined by a generalized linear model to assess the difference between the curves. Error bars, SD from mean. (**B**,**D**,**E**) *p* value, One-way ANOVA with Dunnett’s correction. (*, *p* < 0.05; **, *p* < 0.001).

To expand our findings in NHSCs to a second primary cell culture, we isolated mouse embryonic fibroblasts from C57BL/6N wildtype and *Rabl6* knockout (KO) mice [19]. Two different *Rabl6* knockout MEF cultures were generated from separate embryos and both displayed reduced proliferation compared to the wildtype culture (Figure 4A). Likewise, flow cytometric analyses of DNA content showed a reduced percentage of *Rabl6* KO MEFs in S phase relative to wildtype MEFs (Figure 4B). 

As the cultures were sequentially passaged, MEFs lacking *Rabl6* displayed higher p16 expression at an earlier passage compared to the later induction observed in wildtype counterparts (Figure 4C). Due to their remarkably slow growth, it was difficult to obtain sufficient *Rabl6* KO MEFs for Western detection of most proteins, including RB1. Therefore, we examined RB1 phosphorylation by IF. The heightened p16 expression in low passage *Rabl6* KO MEFs strongly correlated with decreased phosphorylation of RB1 (S807/811) in those cells (Figure 4D). In agreement, early passage *Rabl6* KO MEFs underwent premature senescence relative to wildtype MEFs (Figure 4E). These data further support a role for RABL6A in regulating the senescence of primary cells. 

## 3. Discussion

Proper regulation and maintenance of Schwann cells is critical to the homeostasis of the peripheral nervous system [1]. However, activated oncogenic signaling can lead to aberrant cell cycle reentry and ultimately result in their immortalization and tumor formation. Schwann cell derived tumors are most prominent in the context of NF1, where patients develop benign neurofibromas that can transform into painful and deadly MPNSTs. Therefore, it is crucial that we understand the key signaling pathways that control Schwann cell biology and facilitate tumor formation. Here, we identify RABL6A as a new regulator of Schwann cell senescence. 

This study focused on the effects of RABL6A downregulation in NHSCs and found its loss provoked a premature, stress-induced senescence. That phenotype was shown to be mechanistically dependent on RB1 activation as RB1 loss largely rescued the effects of RABL6A depletion. A similarly reduced proliferative, senescent phenotype was observed in genetically altered MEFs lacking *Rabl6*. The RABL6A-RB1 link in NHSCs is compelling since RB1 inactivation plays such a prominent role in driving MPNST pathogenesis. While the *RB1* gene is rarely altered in these cancers, the RB1 protein is disabled by cyclin E, CDK2 and/or CDK4/6 upregulation as well as by loss of the CDK inhibitors, p27 and p16INK4a, in essentially all MPNSTs [11,20,21]. Our data here establish a critical role for RABL6A in Schwann cell proliferation and senescence, suggesting its dysregulation could be a relatively early event in MPNST development.

In MPNSTs, RABL6A levels are exceedingly high [12]. Therefore, it will be interesting in future studies to examine the effects of elevated RABL6A on normal Schwann cell proliferation, immortalization, and transformation. We speculate that upregulation of RABL6A in non-transformed cells may initially trigger OIS similar to hyperproliferative signals from activated Ras. In most cells, that arrest would be permanent and prevent subsequent transformation. However, it is well established that the selective pressure exerted by OIS ultimately causes a fraction of cells to evade the arrest over time, initiating a cascade of events culminating in malignant transformation [1,2]. Thus, RABL6A overexpression may provoke a transient OIS in primary Schwann cells that would likely be overridden by concomitant inactivation of tumor suppressors (e.g., RB1, p16INK4a, ARF, and/or neurofibromin) or overexpression of activated oncogenes (e.g., Ras). In fact, human MPNSTs display all of those alterations together, namely activated Ras signaling due to neurofibromin loss along with p16INK4a loss (RB1 inactivation), ARF loss (p53 activation), and RABL6A upregulation [12,21].

In conclusion, this study investigated how RABL6A loss influenced the biology of non-transformed, primary human Schwann cells. The results establish a new role for RABL6A in regulating Schwann cell senescence, predicting a potential role in their immortalization and transformation to MPNSTs. Therefore, it is possible that intermediate precursor lesions (ANNUBPs) would benefit from RABL6A pathway inhibition and induction of cellular senescence. Indeed, we observed upregulated levels of RABL6A in ANNUBPs, one of the few molecular changes associated with those lesions besides *NF1* and *INK4A/ARF* loss [12]. Preventing ANNUBP transformation to MPNSTs, which could potentially be accomplished using targeted combination therapies against RABL6A effectors like CDKs and MEK [22], would provide a powerful treatment option to NF1 patients. 

## 4. Materials and Methods

### 4.1. Cell Culture

Primary human Schwann cells (NHSC) isolated from human spinal nerve were purchased from ScienCell Research Laboratories (Cat. #1700) and maintained in Schwann Cell Medium (Cat. #1701), Growth Supplement (Cat. #1752), and 5% FBS. NHSCs were grown on poly-l-lysine (Cat #0413) coated culture vessels and sequentially passaged according to manufacturer’s guidelines by seeding at 5000 cells/cm^2^. The various NHSC passages (P) were binned into Lo (P2–4), Med (P5–7), and Hi (P8–10) for analyses. Mouse embryonic fibroblasts (MEFs) from wildtype and Rabl6m/m C57Bl/6N mice were isolated and cultured as previously described [23]. MEF passages (P) were binned into Lo (P2–4), Med (P5–6), and Hi (P7–8) for analyses. 

### 4.2. RNA Interference, virus Production, and Infection

Human *RABL6A* and *RB1* shRNAs in the pLKO.1 lentiviral vector (Open Biosystems, Huntsville, AL, USA) have been described [13]. Lentiviruses encoding *RABL6A* shRNAs (KD1 and KD2), empty vector (CON), and shRNA RB1 (RB1 KD) were produced by lipofection of viral constructs in HEK293T cells and infected into target cells using established methods [13]. Transduced cells were harvested 3 to 5 days after infection, depending on the assay. For combined silencing of RABL6A and RB1, cells were sequentially infected with RB1 shRNA viruses followed by RABL6A shRNA viruses. 

### 4.3. Cell Proliferation and Senescence Assays

For growth curves, low passage cells were seeded at equal density and cell numbers were manually counted using a hemocytometer at the indicated number of days later. To examine cell viability, manual counts were conducted with Trypan blue (1:1 *v*/*v*), where live cells exclude the dye. To measure DNA synthesis, cells were plated onto chamber slides three days after CON, KD1, or KD2 viral infection. The next day, cells were incubated with 10 umol/L 5-ethynyl-2′-deoxyuridine (EdU) for 18 h. EdU incorporation was detected by staining with Andy Fluor 488 dye (Genecopoeia catalog no. A003). Nuclei were detected with DAPI (Invitrogen ProLong Diamond Antifade Mountant with DAPI, P36932, Carlsbad, CA, USA). Confocal microscopy (Olympus Fluoview FV3000, Shinjuku City, Tokyo, Japan) was performed to capture fluorescence images and quantify EdU positivity from three independent experiments (100 or more cells per sample counted). DNA content (percentage S phase) of low passage MEFs was measured by flow cytometry after propidium iodide staining and analyzed using MOD Fit LT software (Verity Software House, Topsham, ME, USA). For senescence assays, high passage cells were plated onto 6-well plates or chamber slides and the next day stained using the Senescence β-Galactosidase Staining Kit (Cell Signaling Technology #9860, Danvers, MA, USA). DIC microscopy (BioTek Cytation 5 Imaging Reader, Winooski, VT, USA) was performed to capture images. β-Galactosidase positive cells were counted (100 or more cells per sample counted) from three or more independent experiments. 

### 4.4. Antibodies

For Western blotting, antibodies were used according to supplier guidelines. Antibodies included those specific for p-RB1-S807/811 (1:1000, no. 8516) and RB1 (no. 9309 and no. 9313, 1:500) from Cell Signaling Technology; human p16 (ab108349, 1:1000) and GAPDH (1:5000, no. Ab8245) from Abcam; mouse p16 (sc-1207, 1:200) from Santa Cruz Biotechnology; HRP-coupled secondary antibodies (nos. NA934 and NA935, 1:2000) from Sigma. RABL6A antibodies (monoclonal and polyclonal) were produced in the Quelle laboratory [13,24]. For immunofluorescence, antibodies included: p-RB1-S807/811 (no. 8516, 1:1000) from Cell Signaling Technology; p16 (no. Ab108349, 1:100) from Abcam, RABL6A (8D1, 1:10) from Quelle laboratory, and secondary fluorescent-conjugated antibodies (Alexa Fluor 488 and 647, 1:5000). DAPI, to detect nuclei, was co-stained using Invitrogen ProLong Diamond Antifade Mountant with DAPI (P36962). 

### 4.5. Western Blotting

Flash frozen cell pellets were lysed in RIPA buffer (50 mM Tris, pH 8.0, 150 mM NaCl, 1% Triton X-100, 0.1% SDS, 0.5% sodium deoxycholate) containing 1 mM NaF, protease and phosphatase inhibitor cocktails (Sigma, P-8340 & P-0044), and 30 µM phenylmethylsulfonyl fluoride (PMSF). Protein concentrations of extracts were determined by BCA protein assay (ThermoFisher, Cat. #23228). Identical protein equivalents were electrophoresed through polyacrylamide gels. Proteins were transferred from gels onto PVDF membranes (Millipore), which were blocked with 5% non-fat milk or 5% BSA in TBST (Tris-buffered saline containing Tween-20) depending on the antibody. Proteins were detected using HRP-conjugated secondary antibodies and enhanced chemiluminescence (ECL, Amersham, Buckinghamshire, UK). Densitometry quantification was performed using ImageJ (NIH).

### 4.6. Immunofluorescence

Cells were plated on poly-D-lysine-coated chamber slides (Corning Ref 354632) and incubated overnight. Slides were washed once with PBS, fixed for 15 min at room temperature with 16% formaldehyde in PBS, washed 3X with PBS, and blocked (PBS with 5% FBS, 0.3% Triton X-100) for 1 h at room temperature. Slides were rinsed 3X with PBS and incubated at 4C overnight in primary antibody solution (PBS with 1% BSA, 0.3% Triton X-100) using antibodies described above. Slides were then washed with PBS and incubated in secondary antibody solution (Anti-Rabbit IgG, Alexa Fluor 488 and 647) for 1 h at room temperature. Slides were washed 3X with PBS then DAPI staining solution added. Slides were coverslipped and incubated in the dark at room temperature overnight. Confocal microscopy (Olympus Fluoview FV3000) was performed to capture images. Fifty or more cells per sample were counted from 3 or more experiments to quantify results.

### 4.7. Statistics

Western data were imaged by scanning densitometry and quantified by ImageJ (NIH). Values for phosphoproteins were normalized to expression of the total protein, while values for all other proteins were normalized to loading control. Differences in levels of protein phosphorylation or expression were displayed as fold change relative to Lo passage NHSCs or wildtype and vector controls. Quantified data were presented as the mean +/− SD or SEM, as indicated. All *p* values, unless otherwise specified, were obtained by Student’s t-test and adjusted for multiple comparisons using Dunnett’s test or the Bonferroni’s method, as indicated. Overall differences between curves were assessed using generalized linear regressions. An adjusted *p* value less than 0.05 was considered statistically significant.

## Data Availability

Not applicable.
